# A Nomogram for Early Prediction of Inflammation, Catabolism, and Immunosuppression Syndrome in Critically Ill Patients

**DOI:** 10.3390/diagnostics16060918

**Published:** 2026-03-19

**Authors:** Valery Likhvantsev, Levan Berikashvili, Mikhail Yadgarov, Alexey Yakovlev, Artem Kuzovlev

**Affiliations:** Department of Clinical Trials, Federal Research and Clinical Centre of Intensive Care Medicine and Rehabilitology, 25 Petrovka Str., Moscow 107031, Russiaartem_kuzovlev@fnkcrr.ru (A.K.)

**Keywords:** chronic critical illness, inflammation, immunosuppression and catabolism syndrome, PICS, ICS, RICD, predictive model

## Abstract

**Background:** Chronic critical illness (CCI) affects ~7.6% of ICU patients worldwide and is associated with poor outcomes, including 25% in-hospital and 50% one-year mortality. A proposed key mechanism is the inflammation-immunosuppression-catabolism (ICS) triad, which contributes to multiple organ failure and independently increases mortality. Although early identification of ICS could improve risk stratification, no clinically applicable predictive tool currently exists. This study aimed to develop and validate a prognostic nomogram to predict ICS development in ICU (Intensive Care Unit) patients. **Methods:** This real-world analysis used electronic health records from the Russian Intensive Care Dataset (RICD). ICS was defined as C-reactive protein > 20 mg/L, albumin < 30 g/L, and lymphocyte count < 0.8 × 10^9^/L. Variables with >30% missing data were excluded, and remaining missing values were handled by multiple imputation. A Cox proportional hazards model was used to construct the nomogram. Internal validation was performed using an 8:2 training–validation split. **Results:** Among 1963 eligible patients, 540 (27.5%) developed ICS. LASSO (Least Absolute Shrinkage and Selection Operator) regression identified nine significant predictors: age, body mass index, SOFA (Sequential Organ Failure Assessment) and FOUR (Full Outline of UnResponsiveness) scores at admission, pneumonia and anemia at admission, platelet count, total protein, and creatinine. The nomogram showed good discrimination, with C-indices of 0.763 (95% CI: 0.741–0.783) in the training set and 0.735 (95% CI: 0.689–0.784) in the validation set. At the optimal cutoff, sensitivity was 0.75, specificity was 0.63, positive predictive value was 0.43, and negative predictive value was 0.87. **Conclusions:** This study presents the first nomogram for predicting ICS in ICU patients, using nine admission variables to reliably identify low-risk individuals. Further external validation is required.

## 1. Introduction

The phenomenon of chronic critical illness (CCI) represents a growing and formidable challenge within intensive care units (ICU) worldwide [1], constituting a significant public health burden [2,3]. Epidemiological studies report that CCI develops in approximately 7.6% of ICU patients [4] and is associated with a poor prognosis: in-hospital mortality approaches 25%, and one-year mortality reaches 50% [5]. This cohort, classified within the high-need, high-cost patient population, is characterized by a disproportionately high utilization of ICU bed days and healthcare resources. At the same time, important questions regarding the treatment, nutritional support, and rehabilitation of these patients remain largely unresolved [6,7,8].

A critical barrier to therapeutic advancement is the incomplete elucidation of CCI’s underlying pathophysiological mechanisms. A predominant hypothesis involves a triad of inflammation, catabolism, and immunosuppression, referred to in the literature as either PICS or ICS. However, the key distinction lies in their definitions: ICS (Immunosuppression, Catabolism, Inflammation Syndrome) denotes the presence of the physiological state itself, whereas PICS (Persistent Inflammation, Immunosuppression, and Catabolism) incorporates a temporal threshold (such as a length of stay in the ICU exceeding 14 days) to diagnose the persistence of this syndrome [9,10]. ICS is proposed as a key driver of the progressive multiple organ failure observed in CCI patients [9]. Nevertheless, the precise nosological relationship between CCI and ICS remains a subject of active investigation, with conceptual frameworks ranging from ICS as a specific endotype within the heterogeneous CCI population to its role as a primary mechanism of critical illness chronicity [10,11].

Notably, a compelling body of evidence demonstrates that the presence of ICS is robustly associated with adverse clinical outcomes, including increased mortality, independent of its conceptual linkage to CCI [10,12,13,14]. This has driven research to identify early predictors of ICS to enable risk stratification and guide potential preventive interventions [11,13,15,16,17,18]. Despite this, existing data are limited and lack integration into a clinically useful predictive tool.

Therefore, the objective of this study was to develop and validate a prognostic nomogram based on clinical and laboratory risk factors to predict the development of ICS in ICU patients.

## 2. Materials and Methods

### 2.1. Data Sources

This real-world data analysis utilized electronic health records (EHRs) from the Federal Research and Clinical Center of Intensive Care Medicine and Rehabilitology (Russian Intensive Care Dataset, RICD [19,20]). The dataset covered all hospital and intensive care unit (ICU) admissions between December 2017 and July 2023. The local Ethics Committee confirmed that no formal approval was required for this study due to the de-identified nature of the data (Committee decision No. 4/23/2 dated 20 December 2023). The study was conducted in accordance with the TRIPOD checklist (Supplementary Table S1).

### 2.2. Selection Criteria

We extracted patient information from the RICD according to the following inclusion criteria: (1) admitted to the ICU, and (2) age ≥18 years old. Daily ICS presence was determined based on three criteria: C-reactive protein (CRP) levels above 20 mg/L, albumin levels below 30 g/L, and lymphocyte counts under 0.8 × 10^9^/L [21]. Patients were excluded if they met any of the following criteria: (1) ICU stay of less than 24 h, (2) readmission to the ICU, (3) ICS assessment not available; and (4) absence of ICD-10 diagnostic information.

### 2.3. Data Extraction

The data were extracted using SQLite version 3.45.2 and DBeaver software application version 21.1.3. The parameters analyzed included the following: (1) general patient information-sex, age, body mass index (BMI), and whether the patient was transferred from another hospital; (2) severity assessment using sequential organ failure assessment (SOFA), Full Outline of UnResponsiveness (FOUR), Glasgow coma scale (GCS), and comorbidity; and (3) laboratory data-white blood cell (WBC) count, neutrophil and lymphocyte counts, neutrophil-to-lymphocyte ratio (NLR), platelet count, international normalized ratio (INR), albumin, total protein, lactate, alanine transaminase (ALT), aspartate transaminase (AST), creatinine, and CRP. For patients with multiple ICS-related measurements on the same day, the highest CRP and the lowest albumin and lymphocyte counts were analyzed. For patients with ICS, the day of ICS onset was determined; for others, the last day on which ICS assessment was possible was evaluated. Follow-up duration corresponded to the entire ICU stay.

### 2.4. Statistical Analysis and Nomogram Development

The study population was divided into two groups according to their ICS assessment. Data distribution was assessed using the Shapiro–Wilk test. Continuous variables were reported as medians (Me) and interquartile ranges (IQRs), while categorical variables were expressed as frequencies and percentages. The chi-square test and Fisher’s exact test were used for comparing categorical variables. The Mann–Whitney U test (Wilcoxon rank-sum test) was applied for continuous variables. Statistical significance was set at *p* < 0.05 (two-sided).

Significant risk factors were identified through LASSO regression with 10-fold cross-validation and λ optimization (Stata 18.0). Missing data exceeding 5% were handled using the automatic multiple imputation method in IBM SPSS Statistics v. 29.0, while median imputation was applied for variables with less than 5% missing data. Variables with more than 30% missing values were excluded from the analysis.

To develop the prediction model, the cohort was randomly divided into training and validation sets in an 8:2 ratio (internal validation). Variables selected by LASSO regression were incorporated into a Cox proportional hazards model to construct a prognostic nomogram. The time-to-event outcome was defined as days from ICU admission to ICS onset. The ‘nomocox’ command in Stata 18.0 was employed for nomogram development, with manual adjustments for variable ranges and decimals, following established recommendations [22].

### 2.5. Model Performance and Validation

The predictive performance of the nomogram was assessed using Harrell’s C-index and the area under the receiver operating characteristic (AUROC) curve for prognostic indices. Both the C-index and AUROC were evaluated on the training and validation datasets. The 95% confidence intervals for the C-index were estimated via bootstrapping with 1000 resamples. ROC curves were generated using Cox models with prognostic indices derived from the training set, and AUROC values were calculated. The optimal cutoff point for the prognostic index was determined using Youden’s J statistic. Sensitivity, specificity, positive predictive value (PPV), negative predictive value (NPV), and accuracy were evaluated at the selected cutoff point.

The final nomogram was constructed using the complete dataset.

For ROC curve visualization and C-index assessment, Python 3.12.0 was used along with the following modules: ‘pandas’, ‘numpy’, ‘lifelines’, and ‘matplotlib’. All statistical analyses were performed using IBM SPSS Statistics v. 29.0, Stata v. 18.0, and Python 3.12.0.

## 3. Results

### 3.1. Patient Characteristics

**Figure 1:**
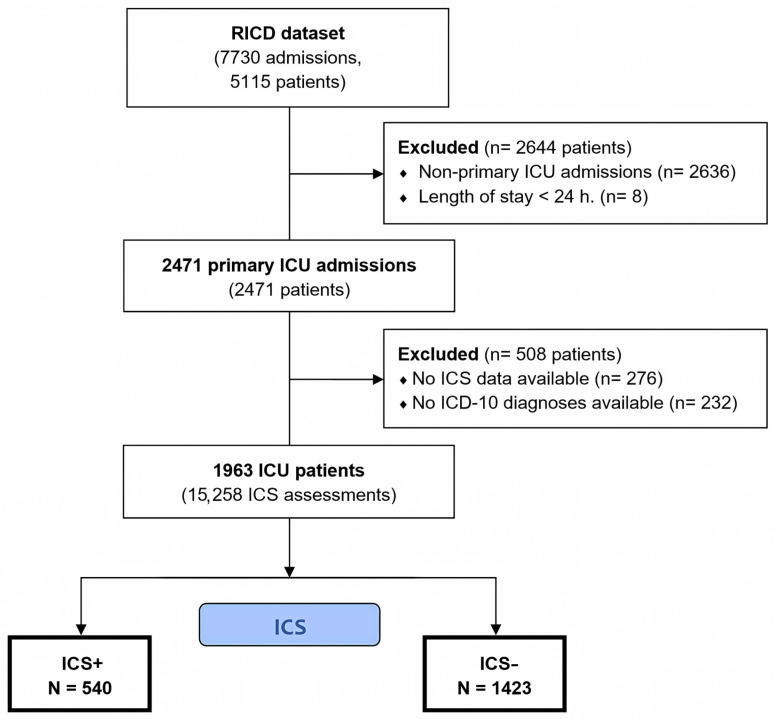
Flowchart of patient selection in the study.

A flowchart of patient selection in the study is presented in Figure 1. After applying the exclusion criteria, a total of 3152 patients were excluded, resulting in an analysis cohort of 1963 patients (55% male). Among these, 540 patients (27.5%) developed ICS. The median age of the cohort was 62 years (IQR 49–73).

Patients who developed ICS were significantly older (median age 69 vs. 60 years, *p* < 0.001) and presented with higher SOFA scores (median 4.0 vs. 2.0, *p* < 0.001) and lower FOUR (median 13.0 vs. 16.0, *p* < 0.001) and GCS scores (median 11.0 vs. 14.0, *p* < 0.001) at admission (Table 1). Pneumonia at admission was more common in ICS patients (46% vs. 32%, *p* < 0.001). Patients with ICS also had a higher prevalence of a history of heart failure (27% vs. 18%, *p* < 0.001) and coronary artery disease (25% vs. 18%, *p* < 0.001).

ICS patients exhibited elevated WBC counts (median 9.1 vs. 8.5 × 10^9^/L, *p* = 0.008), neutrophils (median 7.2 vs. 5.9 × 10^9^/L, *p* < 0.001), NLR (median 8.6 vs. 4.0, *p* < 0.001), INR (median 1.2 vs. 1.1, *p* < 0.001), and creatinine (median 78.8 vs. 75.7 μmol/L, *p* = 0.026), while having lower platelet counts (median 248.0 vs. 290.0 × 10^9^/L, *p* < 0.001), albumin levels (median 28.5 vs. 33.5 g/L, *p* < 0.001), and total protein levels (median 57.6 vs. 63.3 g/L, *p* < 0.001).

### 3.2. Screening for Predictive Factors

**Figure 2:**
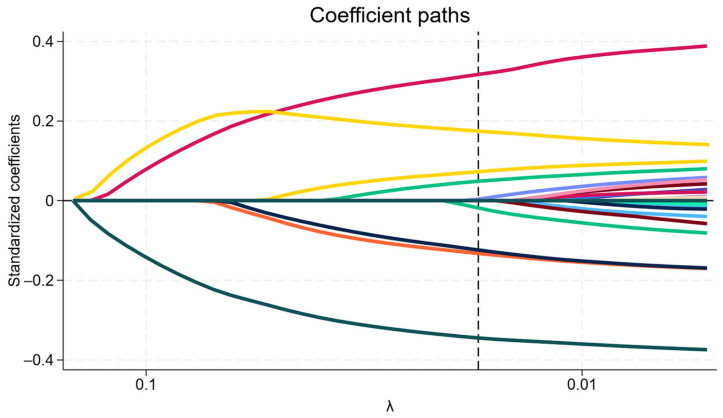
Coefficient paths of selected prognostic variables using LASSO regression. The plot illustrates the coefficient paths of prognostic variables as a function of log(λ) in the LASSO regression model. Each line represents the standardized coefficient of a variable, tracing its value as the penalty parameter (lambda) decreases. The vertical line indicates the value of λ = 0.0173 (λ on a reverse logarithmic scale), selected through 10-fold cross-validation, where the model optimally balances prediction accuracy and model complexity.

LASSO regression was employed to identify significant predictors, and the variation characteristics of these variables are shown in Figure 2. A total of 30 covariates were considered. The LASSO Cox model selected 9 key predictors at the optimal lambda value. This analysis highlighted age, BMI, SOFA and FOUR scores, pneumonia and anemia at admission, platelet count, total protein, and creatinine levels as relevant variables.

A Cox proportional hazards model was subsequently constructed using the variables identified by LASSO regression (Table 2).

### 3.3. ICS Prediction Nomogram Development

**Figure 3:**
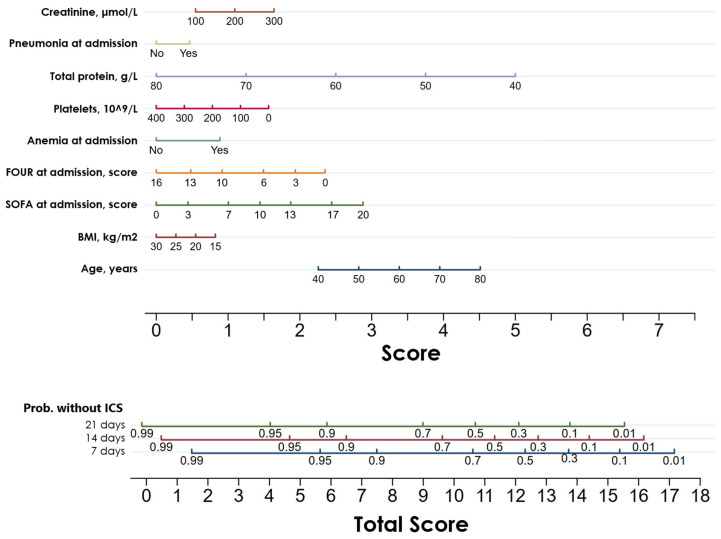
Nomogram to predict the probability of survival without ICS in ICU patients at three time points: 7, 14, and 21 days after ICU admission.

The patients were randomly divided into the training (Ntotal = 1555, NICS = 436, 28.0%) and validation sets (Ntotal = 408, NICS = 104, 25.5%). The proportion of patients with ICS in both datasets was comparable (*p* = 0.305). Based on LASSO regression, a prognostic nomogram was developed, incorporating nine significant predictors (Figure 3). This nomogram was subsequently validated using the validation set.

### 3.4. Validation of the Prediction Nomogram

**Figure 4:**
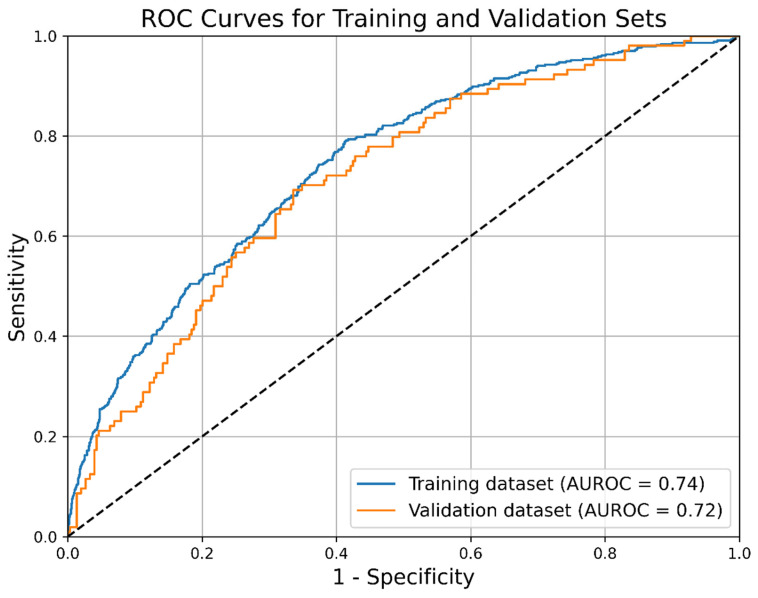
ROC curves for the nomogram. Blue: Training set; Orange: Validation set.

The C-index was 0.763 (95% CI: 0.741–0.783) for the training set, and 0.735 (95% CI: 0.689–0.784) for the validation set. ROC curves are presented in Figure 4. The optimal cutoff point for the prognostic index was −1.02. At this threshold, the model demonstrated a sensitivity of 0.75 (95% CI: 0.71–0.78) and a specificity of 0.63 (95% CI: 0.60–0.65). The PPV was 0.43 (95% CI: 0.40–0.46), while the NPV was 0.87 (95% CI: 0.85–0.89), with an overall model accuracy of 0.66 (95% CI: 0.64–0.68).

## 4. Discussion

### 4.1. Key Findings

In this study, we analyzed data from the Russian Intensive Care Dataset (RICD). Out of the entire database, 1963 patients were selected based on the eligibility criteria, and 540 (27.5%) of these patients developed ICS. Nine significant risk factors were identified using LASSO regression: age, BMI, SOFA and FOUR scores at admission, presence of pneumonia and anemia at admission, platelet count, total protein, and creatinine levels.

A prognostic nomogram based on Cox regression analysis was developed based on these factors, allowing for the prediction of the probability of survival without ICS at 7, 14, and 21 days, following ICU admission. The data were randomly split into training and validation sets in an 8:2 ratio, and model validation was conducted. The C-index for the training set was 0.763 (95% CI: 0.741–0.783), while the C-index for the validation set was 0.735 (95% CI: 0.689–0.784).

At the optimal cutoff point, the model demonstrated a sensitivity of 0.75, specificity of 0.63, PPV of 0.43, and NPV of 0.87. Given the low PPV, the model is better suited for identifying low-risk patients (high NPV) rather than accurately predicting high-risk cases.

### 4.2. Relationship with Previous Studies

Our findings are consistent with several elements of the existing literature on predictors for ICS.

In agreement with our results, Zhang, L. et al. also identified a high admission SOFA score as an independent risk factor for ICS development [17]. Notably, their study further identified pre-existing severe T3 deficiency (<60 ng/dL) as an additional independent predictor, a variable not assessed in our cohort.

In contrast, the study by Nakamura, K. et al. reported male sex and admission levels of creatinine kinase, antithrombin activity, and thrombomodulin as significant predictors [13]. This discrepancy is likely attributable to fundamental differences in the variable sets analyzed, as their investigated predictors differed substantially from ours.

Our results partially align with those of Suzuki, G. et al., who found that transfusion of red blood cells or platelets was an independent risk factor for ICS. This is consistent with our findings of admission anemia and low platelet count as significant predictors [18]. However, they also reported C-reactive protein (CRP) level as a risk factor. We deliberately excluded CRP from our predictive modeling as it is a core component of the systemic inflammatory response inherent to the ICS definition.

Significant overlap exists with the work of Zhong, M. et al., who demonstrated that SOFA score was independent predictor, matching our findings [15]. They also identified low albumin and low lymphocyte count as risk factors. These specific laboratory markers were not included in our primary predictive model as they are integral to the definition of the catabolic and immunosuppressive arms, respectively, of the ICS triad, similar to the rationale for excluding CRP.

Our model consolidates and expands upon previous work by integrating a distinct set of robust, clinically accessible predictors available at ICU admission.

### 4.3. Significance of Study Findings

This study introduces the first validated prognostic model for stratifying the risk of ICS in ICU patients. By integrating nine readily available clinical parameters at admission, the developed nomogram provides a practical tool to shift from the delayed recognition of established ICS to the early identification of high-risk patients, creating a potential window for preventive strategies. Although some hazard ratios (e.g., platelet count and creatinine) appear small when expressed per unit, this reflects the continuous nature of these variables. Their clinical impact is better appreciated when considering meaningful changes: a 50 × 10^9^/L drop in platelets confers an approximately 10% increase in ICS risk, and a 20 μmol/L rise in creatinine corresponds to a 4% increase. Thus, these predictors, while individually subtle, collectively contribute to the model’s risk stratification.

The model’s primary clinical utility is its robust negative predictive value (NPV of 0.87). This allows clinicians to reliably identify a substantial patient subgroup with a low probability of ICS progression, enabling more streamlined care and avoiding unnecessary or potentially harmful interventions. Clinically, this profile suggests that the primary utility of the model lies in its ability to rule out ICS, potentially allowing clinicians to de-escalate monitoring or avoid unnecessary interventions in patients identified as low-risk. Conversely, the modest PPV underscores that patients identified as high-risk by the nomogram should not be automatically labeled as destined to develop ICS, but rather warrant closer clinical observation and further diagnostic evaluation.

In addition to its predictive function, the model reinforces and expands the understanding of factors associated with ICS. It validates the role of admission organ failure (SOFA score) while linking the syndrome to other initial patient characteristics: a diagnosis of pneumonia, which is a known strong driver of systemic inflammation, and lower levels of BMI and total protein, pointing to the influence of pre-existing metabolic and nutritional state.

### 4.4. Strengths and Limitations

This study has several notable strengths. First, it is based on a large, real-world cohort from the RICD, enhancing the generalizability of our findings. Second, to our knowledge, this work presents the first validated prognostic nomogram specifically designed for the early risk stratification of ICS, addressing a significant unmet clinical need. The model utilizes nine routinely available clinical and laboratory parameters, making it pragmatic and immediately applicable at the bedside. Methodologically, the use of LASSO regression for variable selection reduces overfitting, while the robust internal validation process—including temporal split and performance assessment via the C-index and ROC analysis—supports the model’s reliability.

However, this study also has several limitations. First, as a single-center study, the model was validated using internal data, which may limit its generalizability. Future validation using external datasets is needed to confirm the model’s accuracy. Second, due to missing data, some variables (e.g., lactate levels) were excluded, and multiple imputation was applied for some others, which may introduce some uncertainty. Third, the model’s low PPV limits its effectiveness in predicting high-risk patients. Fourthly, formal calibration analysis was not performed, which limits the assessment of the model’s predictive accuracy in estimating absolute risk probabilities. Finally, the proportional hazards assumption was not formally tested, and the absence of external validation, calibration plots, and decision curve analysis represents important methodological constraints that should be addressed in future studies.

## 5. Conclusions

This study developed and internally validated the first prognostic nomogram for stratifying the risk of ICS in ICU patients. The model utilizes nine admission parameters to reliably identify patients with a low probability of ICS development, offering a practical tool for clinical decision-making.

## Figures and Tables

**Table 1:** Initial parameters and disease characteristics between the ICS and non-ICS groups.

Parameters	ICS, *N* = 540	No ICS, *N* = 1423	* p * -Value
Sex, male	280, 52%	808, 57%	0.05 ^1^
Age, years	69.0 (57.0–79.0)	60.0 (45.0–70.0)	**<0.001 ^2^**
BMI, kg/m^2^	* N * = 495, 24.8 (21.5–29.3)	* N * = 1282, 25.2 (21.8–28.7)	0.8 ^2^
Transfer from other hospital	518, 96%	1374, 97%	0.5 ^3^
SOFA at admission, score	* N * = 389, 4.0 (2.0–5.0)	* N * = 981, 2.0 (1.0–4.0)	**<0.001 ^2^**
FOUR at admission, score	*N* = 408, 13.0 (10.0–16.0)	*N* = 1030, 16.0 (13.0–16.0)	**<0.001 ^2^**
GCS at admission, score	* N * = 410, 11.0 (8.0–14.0)	* N * = 1060, 14.0 (10.0–15.0)	**<0.001 ^2^**
Pneumonia at admission	248, 46%	459, 32%	**<0.001 ^1^**
Ischemic stroke	289, 53%	652, 46%	**0.002 ^1^**
Hemorrhagic stroke	81, 15%	236, 17%	0.4 ^1^
Traumatic brain injury	63, 12%	221, 16%	**0.036 ^1^**
Anemia at admission	27, 5.0%	55, 3.9%	0.3 ^3^
Type 2 diabetes	24, 4.4%	81, 5.7%	0.3 ^3^
Cardiovascular disease	10, 1.9%	28, 2.0%	0.9 ^3^
Chronic kidney disease	12, 2.2%	17, 1.2%	0.097 ^3^
Chronic obstructive pulmonary disease	6, 1.1%	8, 0.6%	0.2 ^3^
Myocardial infarction	1, 0.2%	8, 0.6%	0.5 ^3^
Coronary artery disease	134, 25%	254, 18%	**<0.001 ^1^**
Atrial fibrillation	22, 4.1%	47, 3.3%	0.4 ^3^
Arterial hypertension	345, 64%	927, 65%	0.6 ^1^
Coagulopathy	4, 0.7%	3, 0.2%	0.1 ^3^
Inflammatory disorders of the CNS	7, 1.3%	15, 1.1%	0.6 ^3^
Polyneuropathy	4, 0.7%	8, 0.6%	0.7 ^3^
Heart failure	147, 27%	262, 18%	**<0.001 ^1^**
Valvular heart disease	8, 1.5%	8, 0.6%	0.052 ^3^
Mental and cognitive disorders	106, 20%	336, 24%	0.07 ^1^
Polytrauma	16, 3%	45, 3%	0.9 ^3^
Brain disorders	460, 85%	1191, 84%	0.5 ^1^
Malignant tumor	18, 3.3%	36, 2.5%	0.4 ^3^
Laboratory parameters at admission			
WBC, 10^9^/L	9.1 (6.9–11.9)	8.5 (6.6–11.2)	**0.008 ^2^**
Lymphocyte count, 10^9^/L	0.8 (0.6–1.2)	1.4 (1.1–1.9)	**<0.001 ^2^**
Neutrophil count, 10^9^/L	7.2 (5.1–10.2)	5.9 (4.2–8.5)	**<0.001 ^2^**
NLR	8.6 (5.0–13.8)	4.0 (2.6–6.4)	**<0.001 ^2^**
Platelets, 10^9^/L	248.0 (183.0–335.0)	290.0 (226.0–370.8)	**<0.001 ^2^**
INR	1.2 (1.1–1.4)	1.1 (1.1–1.3)	**<0.001 ^2^**
Albumin, g/L	* N * = 470, 28.5 (25.2–32.5)	* N * = 1219, 33.5 (29.3–37.4)	**<0.001 ^2^**
Total protein, g/L	*N* = 515, 57.6 (52.4–63.4)	*N* = 1360, 63.3 (58.0–68.1)	**<0.001 ^2^**
Lactate, mmol/L	* N * = 279, 1.4 (1.0–2.0)	* N * = 602, 1.4 (1.0–1.9)	0.6 ^2^
ALT, U/L	*N* = 513, 26.1 (15.8–45.3)	*N* = 1362, 28.7 (17.7–50.8)	**0.01 ^2^**
AST, U/L	* N * = 513, 30.0 (20.7–45.5)	* N * = 1359, 29.3 (20.7–43.5)	0.3 ^2^
Creatinine, μmol/L	*N* = 515, 78.8 (59.0–109.9)	*N* = 1366, 75.7 (60.8–93.5)	**0.026 ^2^**
CRP, mg/L	* N * = 505, 58.0 (33.2–124.6)	* N * = 1297, 32.7 (9.9–68.9)	**<0.001 ^2^**

^1^—chi-square test with continuity correction; ^2^—Mann—Whitney U test; ^3^—Fisher’s exact test; statistically significant results are highlighted in bold. **Abbreviations**: ICS, inflammation, catabolism, and immunosuppression; ICU, intensive care unit; BMI, body mass index; SOFA, Sequential Organ Failure Assessment; FOUR, Full Outline of UnResponsiveness; GCS, Glasgow coma scale; MV, mechanical ventilation; WBC, white blood cell count; NLR, neutrophil-to-lymphocyte ratio; INR, International Normalized Ratio; CRP, C-reactive protein; CNS, central nervous system; ALT, alanine transaminase; AST, aspartate transaminase.

**Table 2:** Cox regression to predict ICS based on LASSO regression.

Variables	HR (95% CI)	Coeff (β)	*p*-Value
Age, years	1.02 (1.02–1.03)	0.022	<0.001
BMI	0.98 (0.96–0.99)	−0.021	0.009
SOFA at admission, score	1.06 (1.01–1.11)	0.056	0.022
FOUR at admission, score	0.94 (0.91–0.98)	−0.058	0.001
Anemia at admission	1.41 (0.95–2.09)	0.345	0.085
Platelets count	0.998 (0.998–0.999)	−0.002	<0.001
Total protein	0.95 (0.94–0.96)	−0.049	<0.001
Pneumonia at admission	1.199 (1.002–1.437)	0.182	0.048
Creatinine	1.002 (1.001–1.004)	0.002	0.005

**Abbreviations**: ICS, inflammation, catabolism, and immunosuppression; BMI, body mass index; SOFA, Sequential Organ Failure Assessment; FOUR, Full Outline of UnResponsiveness; HR, hazard ratio; CI, confidence interval.

## Data Availability

The original contributions presented in this study are included in the article/Supplementary Materials. Further inquiries can be directed to the corresponding author.

## Supplementary Materials

The following supporting information can be downloaded at: https://www.mdpi.com/article/10.3390/diagnostics16060918/s1, Table S1. Tripod Checklist: prediction model development and validation.

## Abbreviations

The following abbreviations are used in this manuscript:

ALT	Alanine transaminase
AST	Aspartate transaminase
AUROC	Area Under the Receiver Operating Characteristic Curve
BMI	Body Mass Index
CCI	Chronic Critical Illness
CI	Confidence Interval
CNS	Central Nervous System
Cox	Cox proportional hazards model
CRP	C-reactive protein
EHR	Electronic Health Records
FOUR	Full Outline of UnResponsiveness
GCS	Glasgow Coma Scale
HR	Hazard Ratio
IBM	International Business Machines
ICD-10	International Classification of Diseases, 10th Revision
ICU	Intensive Care Unit
ICS	Inflammation, Catabolism, and Immunosuppression Syndrome
INR	International Normalized Ratio
IQR	Interquartile Range
LASSO	Least Absolute Shrinkage and Selection Operator
Me	Median
MV	Mechanical Ventilation
NLR	Neutrophil-to-Lymphocyte Ratio
NPV	Negative Predictive Value
PICS	Persistent Inflammation, Immunosuppression, and Catabolism Syndrome
PPV	Positive Predictive Value
RICD	Russian Intensive Care Dataset
ROC	Receiver Operating Characteristic
SOFA	Sequential Organ Failure Assessment
SPSS	Statistical Package for the Social Sciences
TRIPOD	Transparent Reporting of a multivariable prediction model for Individual Prognosis Or Diagnosis
WBC	White Blood Cell count
